# Assessment of Alveolar Macrophage Dysfunction Using an *in vitro* Model of Acute Respiratory Distress Syndrome

**DOI:** 10.3389/fmed.2021.737859

**Published:** 2021-09-29

**Authors:** Rahul Y. Mahida, Aaron Scott, Dhruv Parekh, Sebastian T. Lugg, Kylie B. R. Belchamber, Rowan S. Hardy, Michael A. Matthay, Babu Naidu, David R. Thickett

**Affiliations:** ^1^Birmingham Acute Care Research Group, Institute of Inflammation and Ageing, University of Birmingham, Birmingham, United Kingdom; ^2^Institute of Metabolism and Systems Research, University of Birmingham, Birmingham, United Kingdom; ^3^Departments of Medicine and of Anaesthesia, Cardiovascular Research Institute, University of California, San Francisco, San Francisco, CA, United States

**Keywords:** ARDS (acute respiratory disease syndrome), alveolar macrophage (AM), efferocytosis, BAL (bronchoalveolar lavage), Rac1, Rho-associated kinase (ROCK) inhibitor

## Abstract

**Background:** Impaired alveolar macrophage (AM) efferocytosis may contribute to acute respiratory distress syndrome (ARDS) pathogenesis; however, studies are limited by the difficulty in obtaining primary AMs from patients with ARDS. Our objective was to determine whether an *in vitro* model of ARDS can recapitulate the same AM functional defect observed *in vivo* and be used to further investigate pathophysiological mechanisms.

**Methods:** AMs were isolated from the lung tissue of patients undergoing lobectomy and then treated with pooled bronchoalveolar lavage (BAL) fluid previously collected from patients with ARDS. AM phenotype and effector functions (efferocytosis and phagocytosis) were assessed by flow cytometry. Rac1 gene expression was assessed using quantitative real-time PCR.

**Results:** ARDS BAL treatment of AMs decreased efferocytosis (*p* = 0.0006) and Rac1 gene expression (*p* = 0.016); however, bacterial phagocytosis was preserved. Expression of AM efferocytosis receptors MerTK (*p* = 0.015) and CD206 (*p* = 0.006) increased, whereas expression of the antiefferocytosis receptor SIRPα decreased following ARDS BAL treatment (*p* = 0.036). Rho-associated kinase (ROCK) inhibition partially restored AM efferocytosis in an *in vitro* model of ARDS (*p* = 0.009).

**Conclusions:** Treatment of lung resection tissue AMs with ARDS BAL fluid induces impairment in efferocytosis similar to that observed in patients with ARDS. However, AM phagocytosis is preserved following ARDS BAL treatment. This specific impairment in AM efferocytosis can be partially restored by inhibition of ROCK. This *in vitro* model of ARDS is a useful tool to investigate the mechanisms by which the inflammatory alveolar microenvironment of ARDS induces AM dysfunction.

## Introduction

Acute respiratory distress syndrome (ARDS) is an inflammatory pulmonary disorder, which results in hypoxemic respiratory failure. ARDS may develop in response to various insults, with sepsis being the underlying etiology in > 75% of cases (1). Since December 2019, the emergence of severe acute respiratory syndrome coronavirus-2 (SARS-CoV-2) and the ensuing pandemic has vastly increased the incidence of ARDS; initial studies showed that 41.8% of adult patients admitted with SARS-CoV-2 pneumonia developed ARDS (2, 3). Notwithstanding advances in supportive care and ventilation strategies, mortality for moderate to severe ARDS remains at 40–46%, and ARDS-specific treatment options are limited (1). Pharmacological therapies such as dexamethasone and tocilizumab have only been shown to be efficacious in SARS-CoV-2 ARDS (4, 5). We now understand more about how ARDS develops: It requires damage to the alveolar epithelium and endothelium (6), leading to reduced alveolar fluid clearance (7), increased permeability, exaggerated inflammation, and neutrophilic alveolar edema (8). However, the role of alveolar macrophages (AMs) in ARDS pathogenesis is not fully understood.

We have previously shown that AM efferocytosis is impaired in patients with sepsis-related ARDS, compared to a control group of ventilated sepsis patients without ARDS (9). Impaired AM efferocytosis is associated with increased alveolar neutrophil apoptosis and worse clinical outcomes (increased duration of mechanical ventilation and mortality), indicating this defect in efferocytosis plays a key role in the pathogenesis of ARDS (9). Further studies are required to investigate the pathophysiological role of AM dysfunction in ARDS; however, the difficulty in obtaining relevant cells from these patients is a major barrier to undertaking this work. Safety concerns preclude bronchoscopy in many patients with ARDS due to their ventilation status (9). For those patients in whom bronchoscopy can be performed, the bronchoalveolar lavage (BAL) fluid is highly neutrophilic, resulting in a relatively low AM yield, which is often insufficient to undertake all necessary experiments in every patient (9). This significantly limits the effectiveness of ARDS-related AM research.

Owing to these difficulties with isolating AMs from patients with ARDS, we sought to develop an *in vitro* model of ARDS. A previous study has shown that the treatment of monocyte-derived macrophages (MDMs) with ARDS patient BAL impairs macrophage efferocytosis (10). However, AMs are distinct from MDMs in terms of origin, function, and phenotype. Resident AMs develop from yolk sac progenitors at the embryonic stage (11) and can self-renew throughout life (12, 13), independently from monocytes and hematopoietic stem cells. AMs are crucial for maintaining alveolar immune homeostasis; exposure to the external environment requires AMs to finely balance inflammatory responses to infection against resolving functions to prevent immune-mediated tissue damage (14). The intrinsic protolerogenic characteristic of AMs has likely evolved to prevent excessive inflammation in the face of continuous low-level stimulation from a diverse range of foreign particles (15). Therefore, while previous studies utilizing MDMs are useful, they do not constitute the most representative model of ARDS and, therefore, would not be as appropriate to inform a mechanistic investigation. We postulated that by using AMs from lung resection tissue and treating with pooled ARDS patient BAL, we could develop a more accurate model of the AM defect in ARDS. Since ARDS patient BAL contains high concentrations of proinflammatory cytokines, we hypothesized that following treatment AMs will be driven away from a proresolving phenotype and toward a proinflammatory phenotype, which is associated with reduced efferocytosis capacity (16, 17).

Alveolar macrophage expression of the Mer tyrosine kinase (MerTK) receptor may be critical for efferocytosis (16, 18). MerTK signaling *via* phosphatidylinositol 3′-OH kinase (PI3K) results in activation of Rac1, which causes cytoskeletal rearrangement and engulfment of the apoptotic cell (19, 20). AM surface receptors, namely, CD206 and CD163, are also thought to mediate efferocytosis (14). AMs also express signal regulatory protein-α (SIRPα) on their surface, which binds surfactant proteins or CD47 on healthy cells (21). SIRPα signaling activates Rho-associated kinase (ROCK), and phosphatase and tensin homolog (PTEN), which oppose PI3K signaling, resulting in Rac1 inhibition and suppression of efferocytosis (22). The status of these important efferocytosis-related receptors in ARDS AMs remains unknown.

We hypothesized that treatment of lung resection tissue AMs with pooled ARDS patient BAL will recapitulate the defect in efferocytosis we observed *in vivo* and allow us to determine the mechanism by which this defect occurs. Our study had the following aims:

(1) To determine whether treatment of lung resection tissue AMs with ARDS patient BAL can replicate the impaired efferocytosis observed in patients with ARDS.

(2) To determine whether treatment of lung resection tissue AMs with ARDS patient BAL decreases AM expression of MerTK and increases expression of SIRPα.

(3) To determine whether inhibition of ROCK-PTEN signaling can increase AM efferocytosis in this *in vitro* model of ARDS.

## Materials and Methods

### Ethical Approval

Ethical approval was obtained to recruit ventilated sepsis patients with and without ARDS (REC 16/WA/0169) and for the use of lung tissue samples from patients undergoing routine thoracic surgery (REC 17/WM/0272). For patients who lacked capacity, permission to enroll was sought from a personal legal representative following the UK Mental Capacity Act (2005). For patients with capacity, written informed consent was obtained from the patient.

### Patient Recruitment

Invasively ventilated adult patients with ARDS and sepsis were recruited from the intensive care unit of the Queen Elizabeth Hospital, Birmingham, UK, from December 2016 to February 2019, and BAL was collected as previously described (9). Demographic and physiological details of the patients can also be found in this prior publication. BAL fluid was rendered acellular by centrifuging at 500 g for 5 min. Acellular supernatant BAL was then pooled and stored at −80°C before use in this study.

Adult patients who underwent lung lobectomy as part of their clinical treatment plan for malignancy at Birmingham Heartlands Hospital from September 2017 to July 2019 were also recruited. Recruited patients were never-smokers or long-term ex-smokers (quit > 5 years), with normal spirometry and without airways disease. No patient received chemotherapy before surgery. Following lobectomy, lung tissue resection samples surplus to histopathological requirements were collected.

### Alveolar Macrophage Isolation

Macroscopically normal lung tissue samples were perfused with 0.15 M saline *via* pressure bag by inserting a needle (21-gauge) in bronchioles. When saturated, the tissue was gently massaged to facilitate emptying lavage fluid from the tissue, ready for the next instillation. This process was repeated until the lavage fluid contained <1 × 10^4^ cells/ml (23).

Cells were pelleted from the lavage fluid by centrifugation at 500 g for 5 min. Mononuclear cells were then separated by gradient centrifugation using Lymphoprep (StemCell Technologies, Vancouver, BC, Canada), according to the instructions of the manufacturer. Mononuclear cells were then cultured in RPMI-1640 media supplemented with 10% fetal calf serum (FCS), 100 U/ml penicillin, 100 μg/ml streptomycin, and 2 mM L-glutamine (Sigma-Aldrich, Darmstadt, Germany) at 37°C and 5% CO_2_ for 24 h to allow adherence. After 24-h culture, the wells were washed and media changed, thereby removing non-adherent mononuclear cells (24, 25). AMs were assessed for purity by cytospin (23); AM purity was consistently >95% across all samples.

### Alveolar Macrophage Efferocytosis Assay

The efferocytosis assay was modified from published protocols (26–29). Neutrophils were isolated from the blood of healthy volunteers using Percoll density centrifugation (30) as previously described by our group (31). Neutrophil purity was >96% as assessed by cytospin and viability >97% as assessed by trypan blue exclusion. Neutrophils were suspended in a 5 μM solution of CellTracker™ Deep Red fluorescent dye (ThermoFisher, Waltham, MA, USA) in 10% FCS/RPMI at 4 × 10^6^/ml, then incubated for 30 min at 37°C. Stained neutrophils were centrifuged at 1,500 g for 5 min then resuspended at 2 × 10^6^/ml in serum-free RPMI and incubated at 37°C and 5% CO_2_ for 24 h to allow apoptosis. Flow cytometric assessment of neutrophil apoptosis was performed using a fluorescein isothiocyanate (FITC)-conjugated Annexin V and 7-aminoactinomycin D (7-AAD) apoptosis detection kit (BioLegend, San Diego, CA, USA): mean neutrophil apoptosis of 93% with necrosis of <2% was observed.

Alveolar macrophages were cultured at 2.5 × 10^5^/well in 24-well plates. As a negative control, 5 μg/ml Cytochalasin D (CytoD, Sigma-Aldrich, Darmstadt, Germany) was added for 30 min to inhibit actin filament polymerization required for efferocytosis. Stained apoptotic neutrophils (ANs) were added to AMs at a 4:1 ratio before incubation for 2 h at 37°C. The optimal assay duration of 2 h had previously been determined by time-course experiments. Media was removed and wells washed two times with ice-cold phosphate-buffered saline (PBS) to remove non-adherent/engulfed neutrophils. Cells were harvested using a 5-min TrypLE™ express (ThermoFisher, Waltham, MA, USA) incubation at 37°C, before acquisition using an Accuri C6 flow cytometer and software (BD Biosciences, Franklin Lakes, NJ, USA). AMs and ANs alone were used to set gates for their respective populations on forward and side-scatter plots. ANs alone were used to set a positive gate on the allophycocyanin (APC) plot, which was subsequently used to identify AMs which had engulfed ANs. Minimum 5,000 events gated as AMs were counted for each experimental condition and the percentage of APC^+^ AMs calculated. CytoD-treated AMs (negative control) determined the background fluorescence present due to ANs adhering to the surface of AMs but not being engulfed. This background fluorescence was subtracted from the percentage of APC^+^ AMs in other experimental conditions to give a corrected net efferocytosis index representative of neutrophil engulfment (Supplementary Figure 1). Steps were taken to avoid bias, including drawing gates based on single-cell populations (ANs and AMs) before assessing efferocytosis.

### Alveolar Macrophage Phagocytosis Assay

Alveolar macrophage phagocytosis assays were performed using pHrodo™ red *Escherichia coli* and *Staphylococcus aureus* BioParticle® conjugates (ThermoFisher, Waltham, MA, USA) in a 96-well plate according to the instruction of the manufacturer and as previously described (23). The pHrodo™ beads were prepared according to the instructions of the manufacturer at a final concentration of 1 mg/ml. AMs were seeded at 50,000 cells per well in black well, clear bottomed 96-well plates, and cultured overnight. For negative control wells, 5 μg/ml CytoD (Sigma-Aldrich, Darmstadt, Germany) was added for 30 min. A total of 50 μl of pHrodo bead suspension was added per well and incubated for 6 h at 37°C. After 6 h, cells were washed three times with PBS before adding 100 μl fresh PBS. Fluorescence was measured using a microplate reader (Synergy 2, Bio-Tek, Winooski, VT, USA) set at the excitation/emission spectra of pHrodo™ red dye: 560/585 nm. The negative control (cytochalasin D treated) AMs were used to determine the background fluorescence present due to stained pHrodo™ red BioParticles® adhering to the outside of macrophages but not being engulfed. This background fluorescence value was subtracted from fluorescence values of other experimental conditions, to give the corrected net fluorescence value the representative of phagocytosis. Phagocytosis results were expressed as fold change in relative fluorescence unit from untreated AMs.

### Use of Alveolar Macrophages in an *in vitro* Model of ARDS

Bronchoalveolar lavage from 14 recruited patients with sepsis-related ARDS was rendered acellular by centrifugation and then pooled. The acellular pooled BAL was mixed in a 1:1 ratio with 10% FCS/RPMI. To elicit functional changes associated with ARDS, AMs were treated with this 50% ARDS BAL mixture. AMs were also treated with a 1:1 mixture of 0.9% saline and 10% FCS/RPMI, as vehicle control (VC). Other treatments given in conjunction with 50% ARDS BAL or saline included 200 nM Y-27632 dihydrochloride (Rho-associated protein kinase inhibitor, Apexbio, Houston, TX, USA), 2 μM SF1670 (phosphatase and tensin homolog inhibitor, Selleckchem, Houston, TX, USA), and dimethyl sulfoxide (VC for Y-27632 and SF1670, Sigma-Aldrich, Darmstadt, Germany) at a 1:50,000 dilution. ROCK and PTEN inhibitor treatment doses were determined by dose response on untreated AM efferocytosis (Supplementary Figure 2). Other treatments not combined with 50% ARDS BAL or saline included 50 ng/ml interferon-γ (IFN-γ, Peprotech, UK), 1 μg/ml ultrapure lipopolysaccharide (LPS, Invitrogen, Waltham, MA, USA), 40 ng/ml interleukin-4 (IL-4, Peprotech, UK), and 40 ng/ml IL-13, (Peprotech, UK). Previous studies have shown that macrophage treatment with IFN-γ and LPS can induce a proinflammatory phenotype, whereas IL-4 and IL-13 treatment can induce a proresolving phenotype (17). Cytokine concentrations were based on published methods (32). The 1 μg/ml dose of LPS was based on the lowest dose required to elicit tumor necrosis factor-α (TNFα) production from AMs (Supplementary Figure 3). Efferocytosis, phagocytosis, apoptosis/viability, and RNA extraction for gene expression were performed 24 h after treatment with 50% ARDS BAL. Phenotyping was performed 48 h after treatment. AM apoptosis and viability were assessed using a flow cytometric apoptosis detection kit (BioLegend, San Diego, CA, USA).

### Flow Cytometric Assessment of AM Surface Markers

Alveolar macrophages were labeled with the following antihuman antibodies or their isotype controls: CD206-APC, CD80-PE, CD163-FITC, Mer-APC, and SIRPα-FITC (see Supplementary Table 1). Surface marker expression was assessed by an Accuri C6 flow cytometer and software (BD Biosciences, Franklin Lakes, NJ, USA). AM population was gated on forward and side-scatter plot. The median fluorescence intensity (MFI) in relevant channels from isotype control AMs was subtracted from the MFIs of stained AMs, to give the net MFI for each antibody fluorophore. Results presented as fold change-corrected MFI, as a measure of change in cell surface expression, compared to VC (50% saline).

### Assessment of AM Gene Expression

RNA was isolated from AMs using Nucleospin RNA kits (Machery-Nagel, Düren, Germany) as per the instructions of the manufacturer. RNA quantity was assessed with the NanoDrop 2000 UV-Vis Spectrophotometer (ThermoFisher, Waltham, MA, USA). One-Step Quantifast Probe RT-PCR Kits (Qiagen, Hilden, Germany) were used to assess gene expression with a CFX384 Touch Real-Time PCR Detection System (BioRad, Hercules, CA, USA). Taqman® gene expression assays (ThermoFisher, Waltham, MA, USA) were purchased for 18S on VIC-MGB (ref 4318839) and RAC1 on FAM-MGB (Hs01025984_m1). PCR conditions were used as per the recommendation of the manufacturer. Triplicate data were analyzed using CFX Maestro software (BioRad, Hercules, CA, USA). Relative quantification of target gene mRNA was calculated relative to expression of 18s endogenous control gene.

### Bronchoalveolar Lavage Cytokine and Protein Quantification

Inflammatory cytokine (IL-6, IL-8, TNF-α, IL-1β, macrophage chemoattractant protein-1, IL-10, IL-1ra, and vascular endothelial growth factor) content of pooled patient BAL fluid was measured by a commercially available custom Magnetic Luminex® Performance Assay (R&D Systems, UK) as per the instructions of the manufacturer. Protein concentration in pooled patient BAL fluid was measured using the Pierce™ BCA (Bicinchoninic Acid) Protein Assay Kit (ThermoFisher Scientific, Waltham, MA, USA) as per the instructions of the manufacturer.

### Statistical Analysis

Data were analyzed using Prism 8 software (GraphPad, San Diego, CA, USA). Parametric data are shown as mean and SD. Non-parametric data are shown as the median and interquartile range (IQR). Differences between continuously distributed data were assessed using Welch's *t*-tests for parametric data or Mann–Whitney tests for non-parametric data. Differences between non-parametric paired data were assessed using Wilcoxon matched-pairs signed-rank test. Differences between three or more unpaired parametric data sets were assessed using ANOVA followed by Dunn's multiple comparison tests. Differences between three or more paired parametric data sets assessed using the repeated measures ANOVA followed by Tukey's multiple comparison tests. Two-tailed *p*-values of ≤ 0.05 were considered significant.

## Results

### Patient Characteristics

Samples of BAL from the first 14 patients with sepsis-related ARDS recruited to a previous study (9) were pooled and used to treat AMs isolated from lung resections. This pooled ARDS patient BAL was characterized with regards to inflammatory cytokine and LPS content (Table 1). AMs were isolated from the lung tissue of 16 patients who underwent lobectomy (mean yield of 9 million AMs per patient). The mean age of lobectomy patients was 70 years (SD = 6.9 years). The male:female split for lobectomy patients was 9:7.

**Table 1:** Characterization of pooled ARDS patient BAL.

**Characterization of pooled ARDS patient BAL**	
IL-6	453 pg/ml
IL-8	4,268 pg/ml
IL-1β	98 pg/ml
IL-1ra	3,023 pg/ml
IL-10	6 pg/ml
TNF-α	3 pg/ml
VEGF	209 pg/ml
MCP-1	1,045 pg/ml
IFN-γ	0 pg/ml
LPS	57 pg/ml
Total protein	2.89 mg/ml

*IFN-γ, interferon-γ; IL, interleukin; TNF-α, tumor necrosis factor-α; MCP-1, macrophage chemoattractant protein-1; LPS, lipopolysaccharide; VEGF, vascular endothelial growth factor*.

### ARDS BAL Treatment of Alveolar Macrophages Impairs Efferocytosis and Preserves Bacterial Phagocytosis

**Figure 1:**
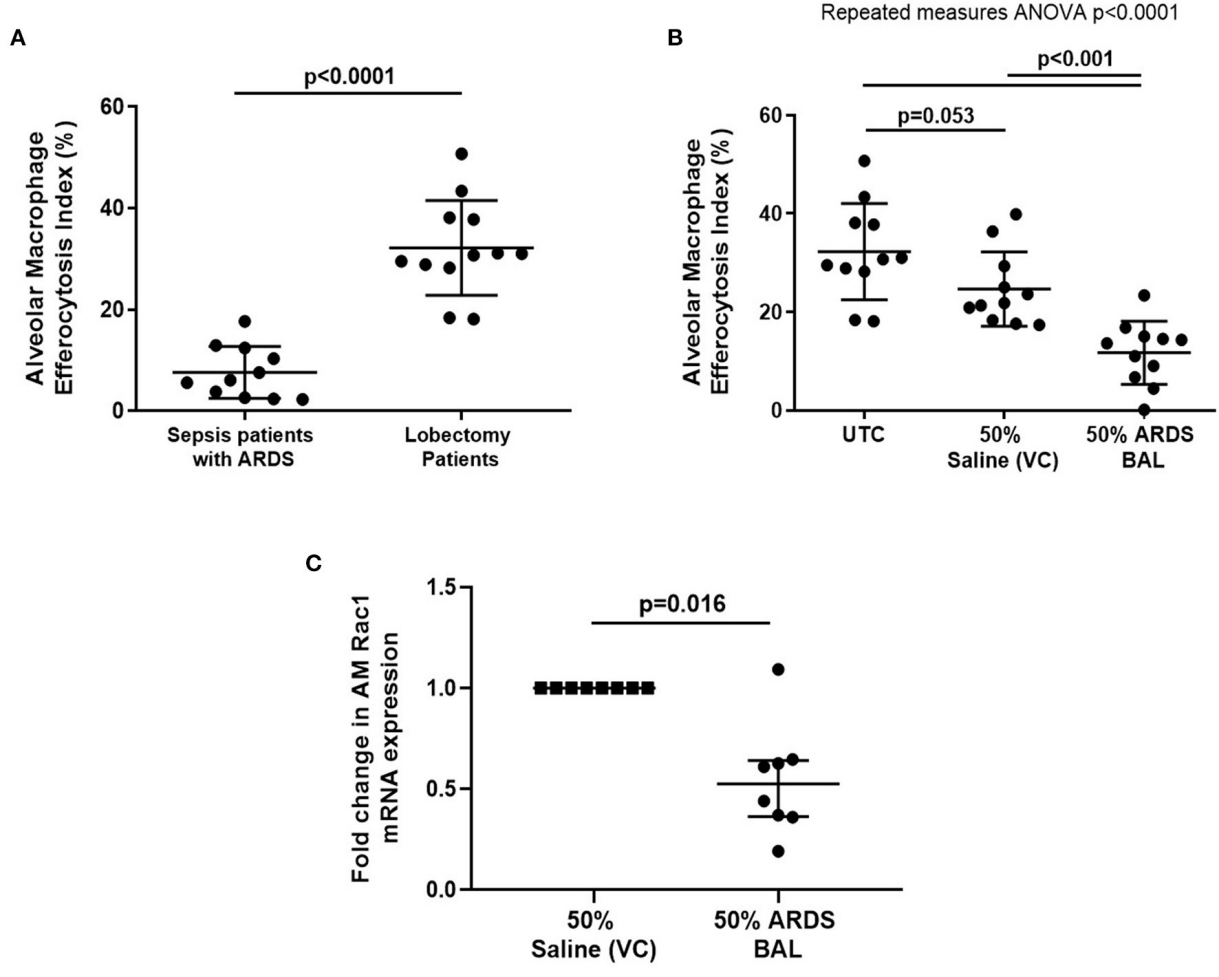
Effect of pooled ARDS BAL treatment on lobectomy patient alveolar macrophage efferocytosis. **(A)** Alveolar macrophages (AMs) from sepsis patients with ARDS have significantly reduced efferocytosis index compared to AMs from lobectomy patients (means 7.6 vs. 32.2%, *p* < 0.0001). Statistical analysis by Welch's *t*-test, *n* = 11–12. **(B)** UTC, Untreated control (cultured in RPMI + 10% FBS); VC, vehicle control (50% saline). Effect of ARDS BAL treatment on lobectomy patient AM efferocytosis. Treatment with 50% ARDS BAL significantly reduced lobectomy patient AM efferocytosis compared to VC treatment (mean of differences 13.0%, *p* = 0.0006) and UTC (mean of differences 20.6%, *p* = 0.0009). Treatment of AMs with VC did not affect efferocytosis compared to UTC (mean of differences 7.6%, *p* = 0.053). Statistical analysis by the repeated measures ANOVA and Tukey's multiple comparisons tests, *n* = 11 for all groups. **(C)** Effect of ARDS BAL treatment on Rac1 gene transcription in lobectomy patient AMs. Data are shown as fold change in AM Rac1 mRNA expression from 50% saline treatment. Statistical analysis by the Wilcoxon matched-pairs signed-rank test, *n* = 8. VC, vehicle control (50% saline). Treatment with 50% ARDS BAL significantly reduced Rac1 mRNA expression in lobectomy patient AMs, compared to VC treatment (median of differences 0.48, *p* = 0.016). Error bars are shown as mean and SD. ARDS, acute respiratory distress syndrome; BAL, bronchoalveolar lavage; FBS, fetal bovine serum.

Alveolar macrophage efferocytosis was impaired in sepsis patients with ARDS compared to lobectomy patients (Figure 1A, mean 7.6% [SD = 5.1] vs. 32.2% [SD = 9.4], *p* < 0.0001). We established an *in vitro* model of ARDS, by treating lung resection tissue AMs with pooled ARDS BAL to induce AM dysfunction. ARDS BAL or saline VC treatment did not affect AM apoptosis or viability compared to standard culture (Supplementary Figure 4). Treatment of AMs with ARDS BAL reduced efferocytosis compared to VC treatment (Figure 1B, mean 11.7% [SD = 6.4] vs. 24.7% [SD = 7.6], *p* = 0.0006). Treatment of AMs with ARDS BAL reduced Rac1 mRNA expression compared to VC treatment (Figure 1C, median of differences 0.48, *p* = 0.016), which supports our finding that ARDS BAL inhibits AM efferocytosis.

**Figure 2:**
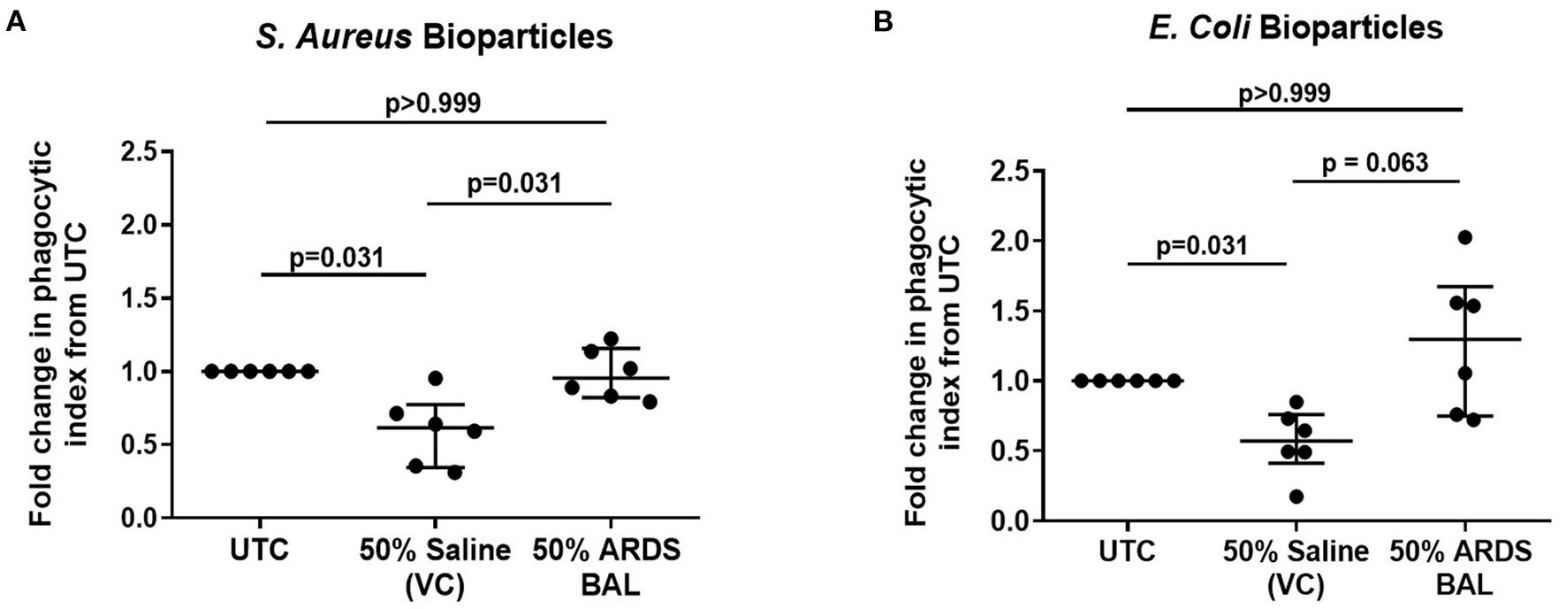
Effect of pooled ARDS BAL treatment on lobectomy patient alveolar macrophage phagocytosis. Alveolar macrophage (AM) phagocytic index in lobectomy AMs treated with 50% ARDS BAL. Data corrected to fold change in the phagocytic index from untreated control (UTC—cultured in RPMI + 10% FBS). Statistical analysis by Wilcoxon matched-pairs signed-rank test, *n* = 6 for all groups. VC, vehicle control (50% saline). VC values are non-identical in graphs C and D. Data are shown as median and interquartile range. ARDS BAL or saline VC-treated AMs receive half the volume of culture media and FCS compared to UTC AMs. **(A)** Treatment of AMs with 50% saline VC reduced phagocytosis of *S. aureus* bioparticles (median of differences −0.38, *p* = 0.031). Treatment with 50% ARDS BAL caused a significant increase in AM phagocytosis of *S. aureus* bioparticles compared to VC (median of differences 0.32, *p* = 0.031). **(B)** Treatment of AMs with 50% saline VC reduced phagocytosis of *E. coli* bioparticles (median of differences −0.43, *p* = 0.031). No significant difference in AM phagocytosis of *E. coli* bioparticles was observed following treatment with 50% ARDS BAL compared to VC (median of differences 0.59, *p* = 0.063). ARDS, acute respiratory distress syndrome; BAL, bronchoalveolar lavage; UTC, untreated control; FCS, fetal calf serum.

**Figure 3:**
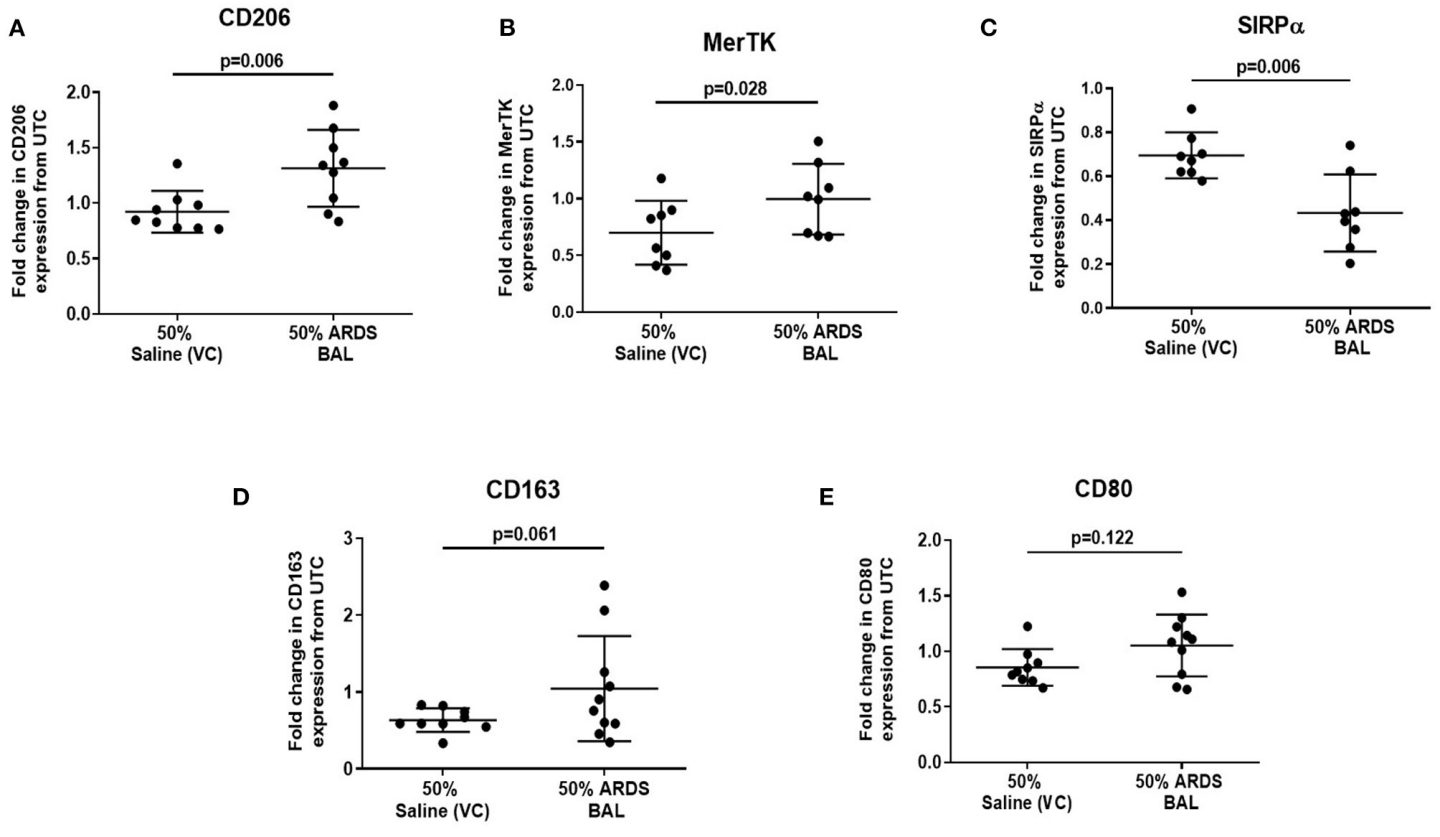
Effect of ARDS BAL treatment on lobectomy patient alveolar macrophage surface-receptor expression. UTC, untreated control (RPMI + 10% FBS); VC, vehicle control (50% saline). Statistical analysis by paired *t*-test, *n* ≥ 8 for all groups. **(A)** Treatment with 50% ARDS BAL significantly increased CD206 expression on AMs compared to VC treatment (mean of differences 0.39, *p* = 0.006), **(B)** 50% ARDS BAL treatment significantly increased MerTK expression on AMs compared to VC treatment (mean of differences 0.30, *p* = 0.028), **(C)** 50% ARDS BAL treatment significantly decreased SIRPα expression on AMs compared to VC treatment (mean of differences −0.26, *p* = 0.006), **(D)** 50% ARDS BAL treatment increased CD163 expression on AMs compared to VC treatment; AM, alveolar macrophage; however, this difference did not reach statistical significance (mean of differences 0.44, *p* = 0.061), and **(E)** 50% ARDS BAL treatment did not significantly change CD80 expression on AMs compared to VC treatment (mean of differences 0.19, *p* = 0.122). ARDS, acute respiratory distress syndrome; BAL, bronchoalveolar lavage; FCS, fetal calf serum; MerTK, Mer receptor tyrosine kinase; SIRPα, signal regulatory protein alpha; AMs, alveolar macrophage.

Treatment of AMs with VC reduced phagocytosis of both *S. aureus* and *E. coli* pHrodo® bioparticles compared to untreated controls (Figures 2A,B, *p* = 0.031). Treatment of AMs with ARDS BAL increased phagocytosis of *S. aureus* pHrodo® bioparticles compared to VC treatment (Figure 2A, median of differences 0.32, *p* = 0.031). There was no difference in AM phagocytosis of *E. coli* pHrodo® bioparticles following ARDS BAL treatment compared to VC treatment (Figure 3B, *p* = 0.063). Thus, ARDS BAL treatment had divergent effects on AM efferocytosis and phagocytosis.

### ARDS BAL Treatment of Alveolar Macrophages Alters Surface-Receptor Expression

Since ARDS BAL treatment of AMs had divergent effects on efferocytosis and phagocytosis, we used this *in vitro* model to investigate the association between AM phenotype and function. Treatment of AMs with ARDS BAL increased expression of CD206 (Figure 3A, mean fold change 0.39, *p* = 0.006) and MerTK (Figure 3B, mean fold change 0.3, *p* = 0.028) compared to VC treatment. Treatment of AMs with ARDS BAL decreased SIRPα expression compared to VC treatment (Figure 3C, mean fold change −0.26, *p* = 0.006). Treatment of AMs with ARDS BAL did not change the expression of CD163 or CD80 compared to VC treatment (Figures 3D,E, *p* > 0.05 for both). These changes in AM surface-receptor expression were incongruent with the observed defect in AM efferocytosis following ARDS BAL treatment.

**Figure 4:**
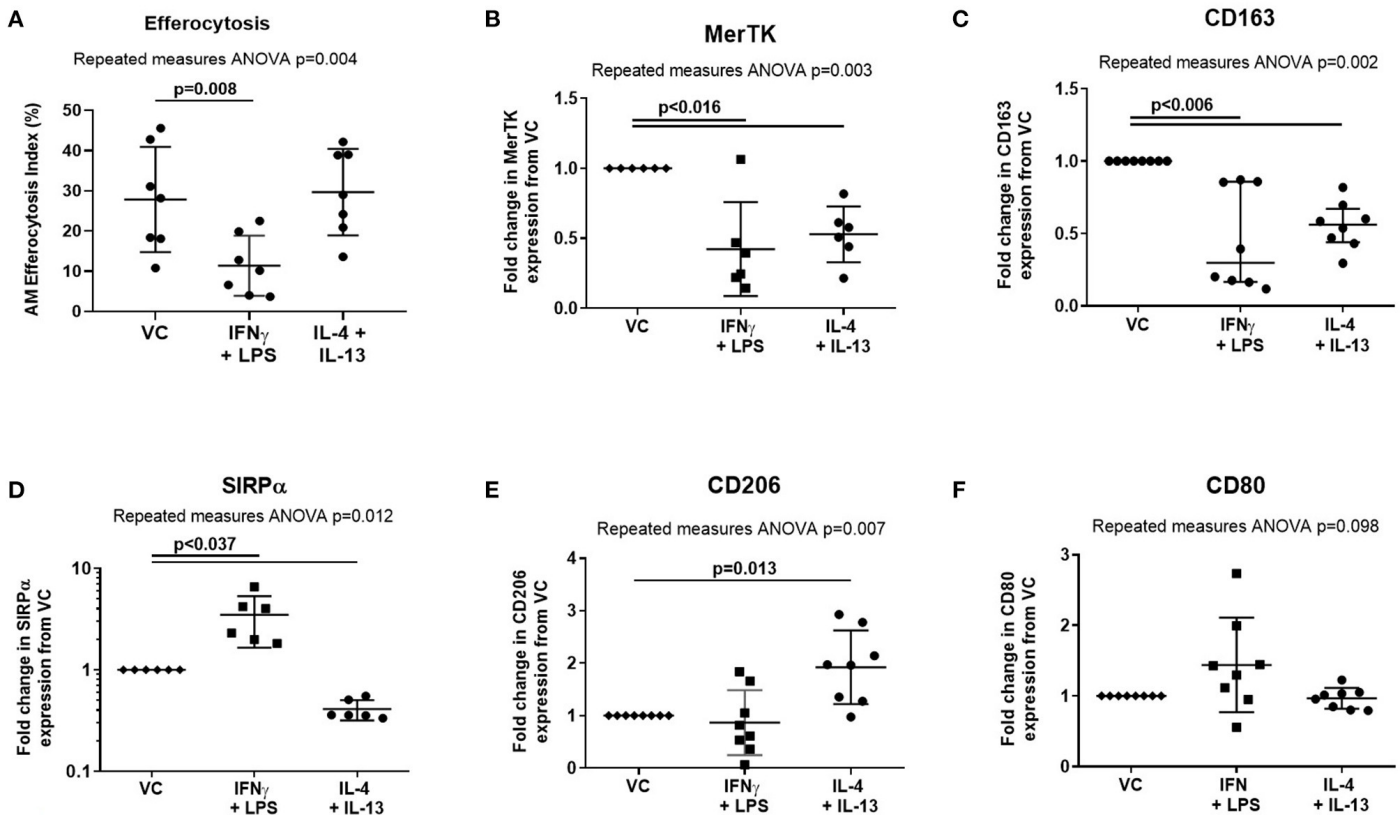
Effect of pro- and anti-inflammatory mediators on alveolar macrophage efferocytosis and surface-receptor expression. VC, vehicle control (distilled water added at 1:500 to RPMI + 10% FBS). IL-4 + IL-13, interleukins 4 and 13 used at 40 ng/ml each; IFN-γ, 50 ng/ml interferon-γ; LPS, 1 μg/ml lipopolysaccharide. **(A)** Data are shown as mean and SD, *n* = 7 for all groups. Cytokine treatment significantly affected alveolar macrophage (AM) efferocytosis (repeated measures ANOVA *p* = 0.004). IFN-γ and LPS treatment significantly reduced AM efferocytosis compared to VC (Dunnett's multiple comparisons test mean difference 16.5%, *p* = 0.008). IL-4 and IL-13 had no significant effect on AM efferocytosis compared to VC (Dunnett's multiple comparisons test mean difference −1.8%, *p* = 0.897). **(B–F)** Statistical analysis by the repeated measures ANOVA with Dunnett's multiple comparison test, *n* = 6–8. Linear *y*-axis was used for all graphs, except SIRPα **(D)** for which a log scale *y*-axis was used. **(B)** Cytokine treatments significantly affected AM surface expression of MerTK (repeated measures ANOVA *p* = 0.003). Compared to treatment with VC, AM surface expression of MerTK was significantly decreased following treatment with IFN-γ + LPS (mean of differences −0.58, *p* = 0.015), and IL-4 + IL-13 (mean of differences −0.47, *p* = 0.004). **(C)** Cytokine treatments significantly affected AM surface expression of CD163 (repeated measures ANOVA *p* = 0.002). Compared to treatment with VC, AM surface expression of CD163 was significantly decreased following treatment with IFN-γ + LPS (mean of differences −0.55, *p* = 0.005), and IL-4 + IL-13 (mean of differences −0.45, *p* = 0.002). **(D)** Cytokine treatments significantly affected AM surface expression of SIRPα (repeated measures ANOVA *p* = 0.012). Compared to treatment with VC, AM surface expression of SIRPα was significantly increased following treatment with IFN-γ + LPS (median of differences 2.48, *p* = 0.036). Compared to treatment with VC, AM surface expression of SIRPα was significantly decreased following treatment with IL-4 + IL-13 (median of differences −0.59, *p* < 0.0001). **(E)** Cytokine treatments significantly affected AM surface expression of CD206 (repeated measures ANOVA *p* = 0.007). Compared to treatment with VC, AM surface expression of CD206 was significantly increased following treatment with IL-4 + IL-13 (mean of differences 0.92, *p* = 0.013). There were no significant changes in CD206 expression following treatment with IFN-γ + LPS (mean of differences −0.13, *p* = 0.777). **(F)** Cytokine treatments did not significantly affect AM surface expression of CD80 (repeated measures ANOVA *p* = 0.098). MerTK, Mer tyrosine kinase receptor; SIRPα, signal regulatory protein-α.

Treatment of AMs with proinflammatory mediators IFN-γ and LPS also impaired efferocytosis (Figure 4A, mean difference 16.5%, *p* = 0.008). However, in contrast to ARDS BAL, proinflammatory mediator treatment decreased expression of both MerTK (Figure 4B, mean fold change −0.58, *p* = 0.015) and CD163 (Figure 4C, mean fold change −0.58, mean fold change −0.55, *p* = 0.005) while increasing expression of SIRPα (Figure 4D, mean fold change 2.48, *p* = 0.036). Proinflammatory mediator treatment had no significant effect on the expression of AM surface markers CD206 or CD80 (Figures 4E,F). Treatment with proresolving mediators IL-4 and IL-13 did not affect AM efferocytosis; however, their effect on AM surface-receptor expression was also assessed: expression of MerTK, CD163, and SIRPα was decreased while expression of CD206 was increased (Figures 4A–E). These findings indicate that the effect of ARDS BAL treatment on AMs cannot solely be explained by changes in surface-receptor expression.

### Rho-Associated Kinase Inhibition Partially Restores Alveolar Macrophage Efferocytosis in an *in vitro* Model of ARDS

**Figure 5:**
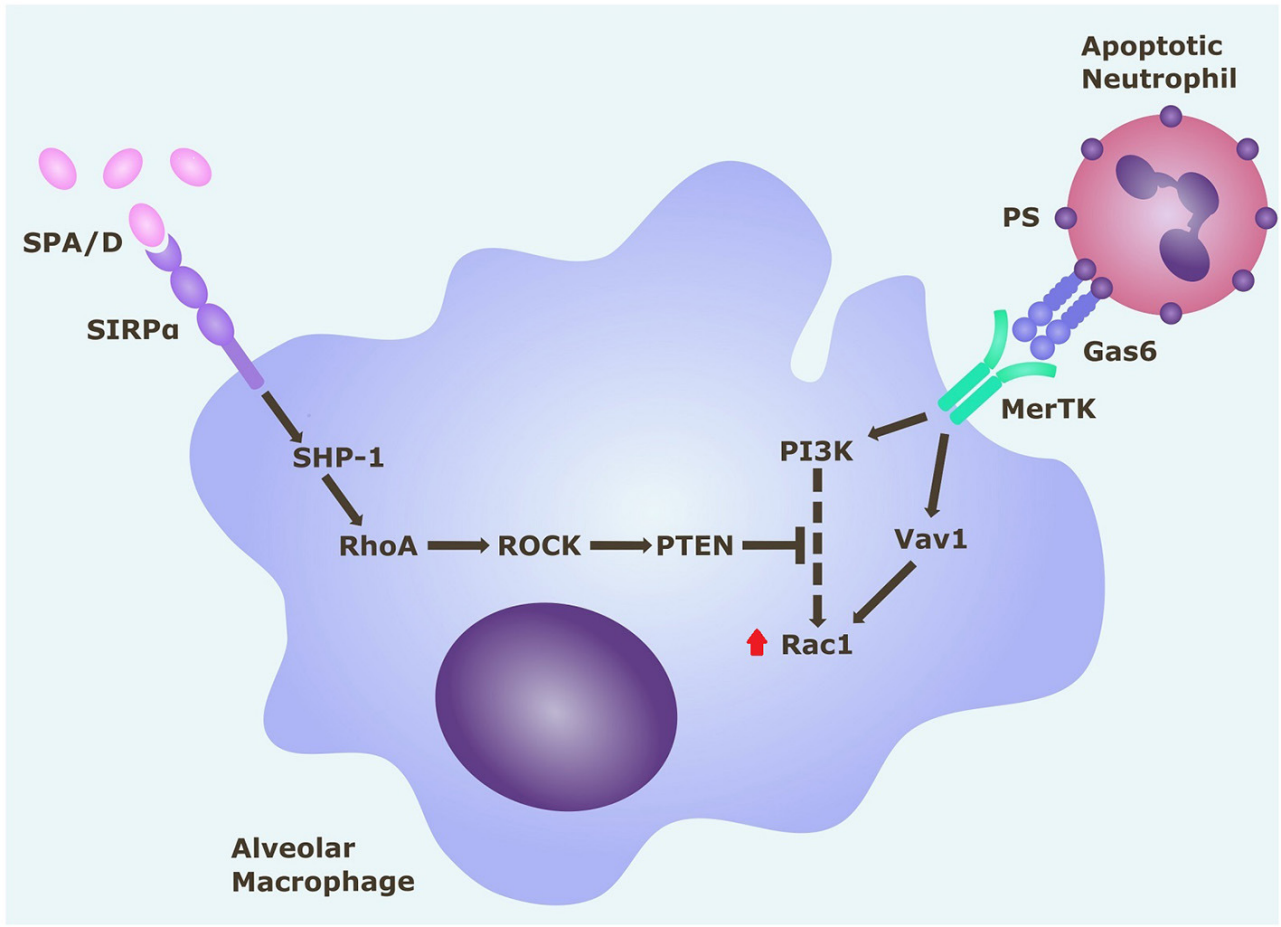
Rac1 intracellular signaling pathways in alveolar macrophages. Alveolar macrophage efferocytosis is regulated by surface receptors MerTK and SIRPα. Gas6 binds to PS on the surface of apoptotic cells. Activation of MerTK by the PS opsonin Gas6 can trigger signaling cascades *via* PI3K and Vav1, which both upregulate Rac1. Activation of Rac1 results in cytoskeletal rearrangement and efferocytosis of the apoptotic cell. Activation of SIRPα by SP-A (or SP-D) triggers a signaling cascade along the SHP1/RhoA/ROCK/PTEN pathway, which inhibits PI3K signaling, and ultimately downregulates Rac1, thereby inhibiting efferocytosis. Gas6, growth arrest specific-6; MerTK, Mer tyrosine kinase receptor; PI3K, phosphatidylinositol 3′-OH kinase; PS, phosphatidylserine; PTEN, phosphatase and tensin homolog; ROCK, Rho-associated kinase; SHP-1, Src homology region 2 domain-containing phosphatase-1; SIRPα, signal regulatory protein-α; SPA/D, surfactant protein A/D.

**Figure 6:**
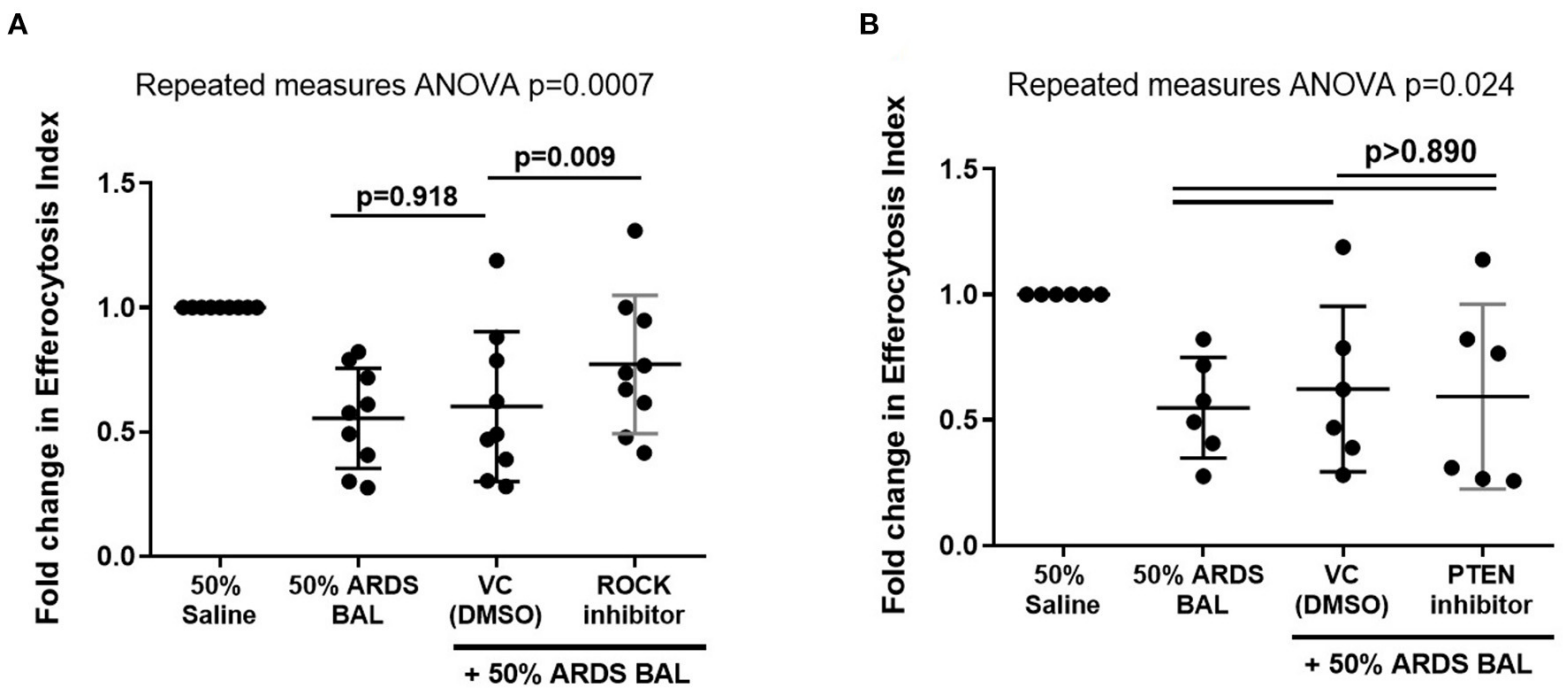
Effect of ROCK and PTEN inhibitors on restoring alveolar macrophage efferocytosis following ARDS BAL treatment. Data are shown as fold change in AM efferocytosis index from 50% saline treatment. ROCK, Rho-associated protein kinase; PTEN, phosphatase and tensin homolog; VC, vehicle control [dimethyl sulfoxide (DMSO) at 1:50,000 dilution]. ROCK inhibitor = 200 nM Y-27632 dihydrochloride. PTEN inhibitor = 2 μM SF1670. Statistical analysis by repeated measures ANOVA with Tukey's multiple comparison test. **(A)** The addition of VC to ARDS BAL mixture had no significant effect on AM efferocytosis (mean of differences 0.05, *p* = 0.918, *n* = 9) compared to ARDS BAL alone. Addition of ROCK inhibitor to ARDS BAL treatment significantly increased efferocytosis compared to treatment with VC + ARDS BAL (mean of differences 0.17, *p* = 0.009, *n* = 9) **(B)** Addition of PTEN inhibitor to 50% ARDS BAL treatment had no significant effect on efferocytosis compared to treatment with VC + ARDS BAL (mean of differences 0.03, *p* = 0.924, *n* = 6).

Since this model effectively replicated *in vitro* the efferocytosis defect in ARDS AMs evident *in vivo*, we next used this model to explore the mechanism driving this defect. Rac1 intracellular signaling pathways are summarized in Figure 5. From this pathway, we identified ROCK and PTEN as potential targets to modify activity. The addition of ROCK-inhibitor to ARDS BAL treatment increased AM efferocytosis compared to treatment with ARDS BAL plus VC (Figure 6A, mean fold change 0.17, *p* = 0.009). The addition of PTEN inhibitor to ARDS BAL treatment did not affect efferocytosis compared to treatment with ARDS BAL plus VC (Figure 6B). ROCK inhibition did not affect AM phagocytosis of *E. coli* or *S. aureus* bioparticles (Supplementary Figure 5). ROCK inhibition also did not affect AM surface marker expression of CD206, CD163, CD80, SIRPα, and MerTK (Supplementary Figure 6).

## Discussion

Herein, we have established a phenotypically and functionally accurate *in vitro* model through which we can model the effects of ARDS on AM function. In this model, ARDS BAL treatment of lung resection tissue AMs induced an impairment in efferocytosis observed in ARDS patients, but preserved AM bacterial phagocytosis. Thus, the inflammatory contents of ARDS BAL do not induce a global impairment in AM function but rather a specific impairment in efferocytosis. The impairment in AM efferocytosis caused by ARDS BAL treatment is not mediated by changes in surface-receptor expression. ROCK inhibition partially restores AM efferocytosis in an *in vitro* model of ARDS. Modulation of the ROCK-PI3K-Rac1 intracellular signaling pathway may offer a therapeutic strategy to upregulate AM efferocytosis in ARDS.

In early ARDS, proinflammatory monocytes migrate to the alveoli and then differentiate into “recruited” AMs (33). A direct correlation was observed between alveolar monocyte influx, the severity of the respiratory failure, and mortality in ARDS (33). Murine models showed that following the initiation of lung injury, the majority of inflammatory cytokines (namely, tumor necrosis factor-alpha, IL-6, and IL-1β) were released by recruited AMs (34). Our study assessed total AM efferocytosis, and did not distinguish between resident and recruited AMs. Therefore, we initially postulated that the decreased AM efferocytosis in ARDS may be due to the polarization of AMs to a proinflammatory phenotype, which is associated with reduced efferocytosis (17). The concentrations of inflammatory cytokines within our pooled ARDS BAL are in keeping with those reported in previous studies (35, 36). We then undertook experiments using the *in vitro* model of ARDS to investigate the association between AM phenotype and function.

Intriguingly, ARDS BAL treatment of AMs increased expression of efferocytosis receptors (CD206 and MerTK) and decreased expression of the antiefferocytosis receptor SIRPα. These phenotypic changes were incongruent with the functional defect in AM efferocytosis induced by ARDS BAL. In comparison, treatment with proinflammatory mediators (IFN-γ and LPS) also decreased AM efferocytosis, but induced SIRPα expression and decreased MerTK expression. Although both ARDS BAL and proinflammatory mediator treatments impaired AM efferocytosis, they had opposite effects on efferocytosis receptor expression. A similar association was observed in cigarette smokers, between decreased AM efferocytosis (37), increased MerTK expression (38), and increased transcription of genes associated with a proresolving phenotype (39). Patients with chronic obstructive pulmonary disease (COPD) also have impaired AM efferocytosis (40) with overexpression of efferocytosis receptors CD206 and CD163 (41). Our data, therefore, suggest that the AM efferocytosis defect induced by ARDS BAL treatment is not mediated by surface-receptor changes.

Strategies to upregulate AM efferocytosis may reduce secondary necrosis of alveolar neutrophils, thereby attenuating inflammation in ARDS. Since *in vitro* ARDS BAL treatment downregulated AM Rac1 gene expression, we sought to upregulate Rac1 expression and restore efferocytosis by inhibiting ROCK and PTEN. The addition of ROCK inhibitor to ARDS BAL treatment partially restored AM efferocytosis function and did not affect bacterial phagocytosis. However, the addition of PTEN inhibitor had no significant effect; this may be because the role of PTEN is less important in antagonizing the PI3K pathway. ROCK inhibitors have been shown to increase efferocytosis in MDMs and AMs from patients with COPD (42). Further studies to investigate the role of the ROCK-PTEN-Rac1 pathway in ARDS AM dysfunction are required. Measurements of Rac1 and PI3K proteins expression in our model, with and without ROCK inhibition, are required to support the hypothesis that Rac1 inhibition is partially responsible for impaired AM efferocytosis in ARDS. ROCK inhibition promotes PI3K signaling, which has multiple effects on a cellular function beyond upregulation of Rac1, namely, proliferation, chemotaxis, and migration (43). ROCK inhibition would have many off-target effects, thereby limiting its therapeutic potential as a strategy to upregulate AM function in ARDS. Existing medications could be tested using the *in vitro* model of ARDS, to determine if they can restore AM efferocytosis, e.g., N-acetylcysteine (44), macrolide antibiotics (45), statins (46), and glucocorticoids (47).

Studies utilizing an *ex-vivo* perfused human lung model of ARDS have shown that extracellular vesicles (EVs) are released following lung injury with *E. coli*; these isolated EVs subsequently mediated inflammatory lung injury when administered to uninjured lungs (48). Murine models of LPS lung injury have shown that EV transfer of microRNA cargo to AMs can increase inflammatory cytokine release (49). Further analysis of ARDS BAL and studies utilizing our *in vitro* model of ARDS are required to determine whether EV transfer of microRNA to AMs may affect intracellular pathways regulating efferocytosis (50).

Our study had some limitations. Due to logistical constraints, efferocytosis assays were undertaken with heterologous neutrophils, as opposed to autologous neutrophils, which would have more accurately reflected the environment *in vivo*. Although unaffected lung tissue was processed, we cannot rule out contamination with tumor-associated macrophages which are characterized by an immunosuppressive phenotype and may exhibit increased efferocytosis (51), which could account for some of the divergent effects observed. Expression of intracellular signaling mediators (e.g., Rac1) was only measured at the mRNA level. To draw definitive conclusions regarding the mechanism of impaired efferocytosis in ARDS, data on the protein expression of these mediators will be required. Ideally, the use of healthy human BAL would be a more appropriate VC instead of saline in this model; however, healthy BAL is a highly limited resource.

Another limitation to our study is that when assessing AM expression of TAM receptors (key mediators of macrophage efferocytosis), only MerTK was investigated (52). We had predominantly focused on MerTK, as this efferocytosis receptor was best characterized in the context of ARDS within the literature (18, 53–55). However, we omitted to investigate other important TAM receptors: Axl and Tyro3 (52). Impairment of the Axl signaling pathway has been associated with decreased AM efferocytosis in asthma (56). We report a contradictory increase in MerTK expression associated with decreased efferocytosis in AMs treated with ARDS BAL; however, this may, in part, be explained if the expression of TAM receptors Axl and/or Tyro3 were decreased. Further studies will be required to investigate this.

Studies have previously shown that the microenvironment can influence AM metabolism, inflammatory response, and gene expression (57, 58). *In vitro* culture of AMs can alter efferocytosis receptor expression profiles (56), therefore undertaking efferocytosis assays directly *in situ* on lung tissue may provide a more accurate representation of *in vivo* AM function (59). For future studies, precision-cut lung slices could be incubated with ARDS BAL before the assessment of efferocytosis directly on lung tissue (60); terminal deoxynucleotidyl transferase dUTP nick end labeling could be used to identify ANs in a double immunofluorescence method (59).

In conclusion, *in vitro* treatment of lung resection tissue AMs with pooled ARDS patient BAL can recapitulate the same functional defect observed *in vivo*. This dysfunction can be partially restored by ROCK inhibition. The *in vitro* model of ARDS is a useful tool to investigate the mechanisms by which the inflammatory alveolar microenvironment of ARDS induces AM dysfunction.

## Data Availability Statement

The original contributions presented in the study are included in the article/Supplementary Material, further inquiries can be directed to the corresponding author/s.

## Ethics Statement

The studies involving human participants were reviewed and approved by Wales Research Ethics Committee 1 (REC 16/WA/0169) and West Midlands - Solihull Research Ethics Committee (REC 17/WM/0272). The patients/participants provided their written informed consent to participate in this study.

## Author Contributions

RM, AS, MM, and DT contributed to the study conception and design. RM, AS, DP, and SL contributed to data acquisition. RM, AS, and DT drafted the manuscript. All the authors contributed to the data analysis and interpretation, critically revised the manuscript for intellectual content, and approved the final version before submission.

## Funding

This work was funded by Medical Research Council grants MR/N021185/1 (RM) and MR/L002736/1 (DT/AS).

## Conflict of Interest

The authors declare that the research was conducted in the absence of any commercial or financial relationships that could be construed as a potential conflict of interest.

## Publisher's Note

All claims expressed in this article are solely those of the authors and do not necessarily represent those of their affiliated organizations, or those of the publisher, the editors and the reviewers. Any product that may be evaluated in this article, or claim that may be made by its manufacturer, is not guaranteed or endorsed by the publisher.
