# Supplementary material for: Assessment of Alveolar Macrophage Dysfunction Using an in vitro Model of Acute Respiratory Distress Syndrome
Source: Front Med (Lausanne). 2021 Sep 29;8:737859. doi: 10.3389/fmed.2021.737859 (PMC8511446; doi:10.3389/fmed.2021.737859)
Supplement: Supplementary file 1 [file Data_Sheet_1.docx]

**Supplemental Information**


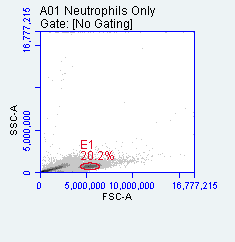

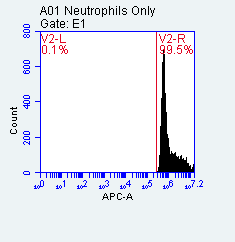


**A**

**
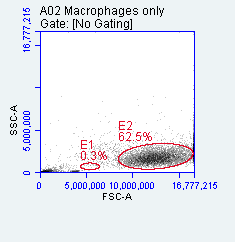

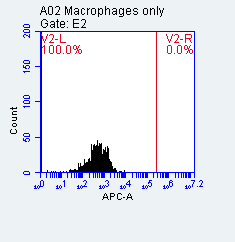
**

**B**

**
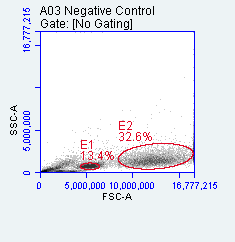

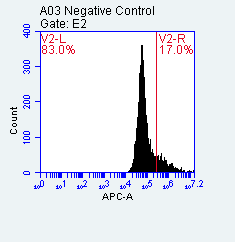
**

**C**

**
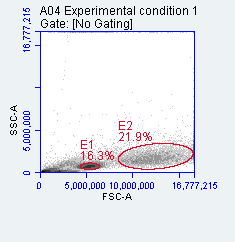

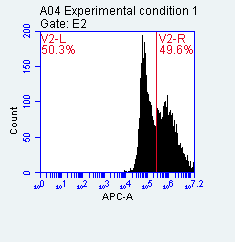
**

**D**

**Supplemental Figure 1: Example flow plots and histograms for alveolar macrophage efferocytosis assay.**

Forward vs side scatter plots and fluorescence histograms of control and test samples used to set up efferocytosis assay. Alveolar macrophages (AMs) used in this example are from the lung resection of a never smoking patient. Doublet discrimination was also performed on all samples but not shown. All fluorescence histograms are of the APC (FL4) channel, which corresponds to the emission spectra of CellTracker^TM^ Deep Red. **A:** Stained apoptotic neutrophils were used to gate neutrophils (E1) on forward vs side scatter plot, and as a positive control used to determine the positive threshold on the APC fluorescence histogram. **B:** Tube containing AMs only used to gate macrophages on forward vs side scatter plot (E2), and used to confirm that AMs alone do not fluoresce above the positive threshold in the APC channel. **C:** Negative control (Cytochalasin D treated AMs incubated with neutrophils) in which efferocytosis has been inhibited. The APC fluorescence histogram has been gated on E2 (macrophage gate). Since efferocytosis has been inhibited, any fluorescence in the APC channel detected above the positive threshold is due to neutrophil adherence to the surface of AMs, and not engulfment. This background fluorescence value is 17%. **D:** Experimental condition (untreated AMs incubated with neutrophils). The APC fluorescence histogram has been gated on E2 (AM gate), and shows that 49.6% of AMs are APC-positive. However, the background fluorescence value of 17% from the negative control must be subtracted to determine the true efferocytosis index. The efferocytosis index for this sample would therefore be calculated as 32.6%.


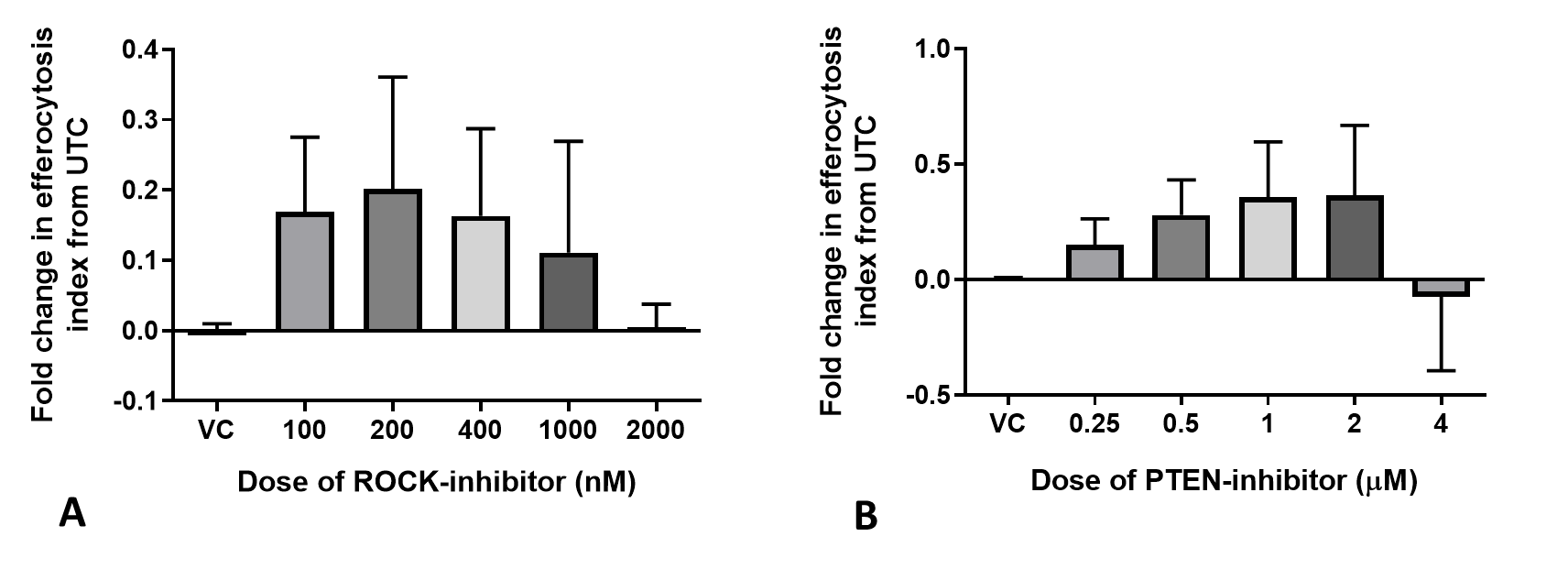


**Supplemental Figure 2: Dose response of ROCK-inhibitor and PTEN-inhibitor on the efferocytosis index of uninjured alveolar macrophages.**

Data shown as mean and standard deviation, n=4 all groups. UTC = Untreated control. VC = Vehicle Control (Dimethyl Sulfoxide [DMSO] at 1:50,000 dilution). ROCK-inhibitor = 200nM Y-27632 dihydrochloride; Rho-associated protein kinase inhibitor. PTEN-inhibitor = 2μM SF1670; Phosphatase and tensin homolog inhibitor. **A:** The dose of ROCK-inhibitor which elicited the greatest fold change in the efferocytosis index of uninjured alveolar macrophages was 200mM, equal to the IC_50_ (50% of the maximal inhibitory concentration) **B:** The dose of PTEN-inhibitor which elicited the greatest fold change in the efferocytosis index of uninjured alveolar macrophages was 2µM, equal to IC_50_.

**Supplemental Figure 3: Dose response of LPS on alveolar macrophage secretion of TNF-α**

LPS = Lipopolysaccaride. TNF-α = Tumour necrosis factor-α. UTC = Untreated control. n=3. LPS only stimulated AM secretion of TNFα at doses ≥ 1µg/ml.


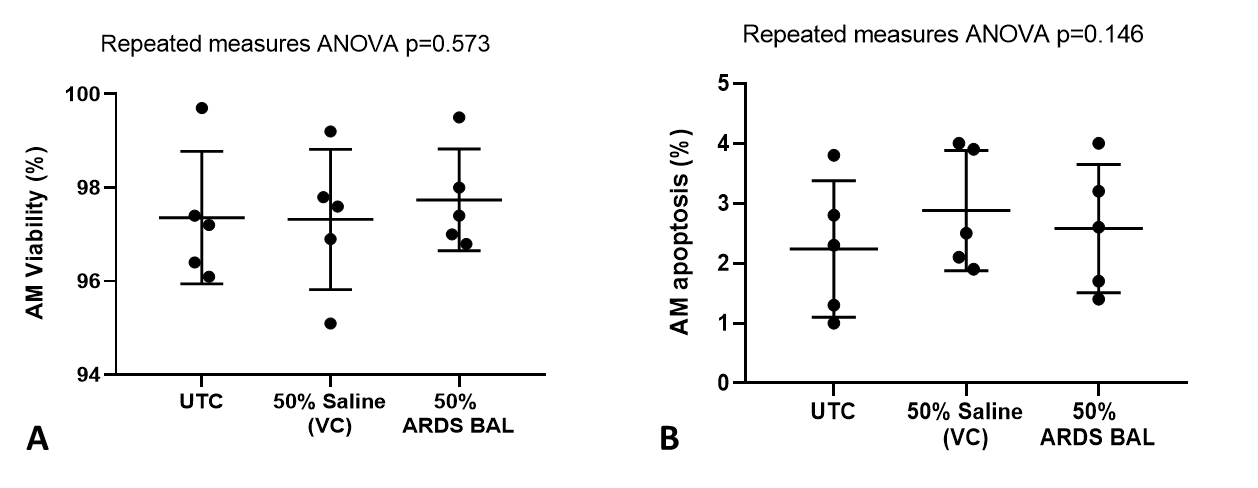


**Supplemental Figure 4: Effect of ARDS BAL on alveolar macrophage viability and apoptosis.**

UTC = Untreated control (RPMI + 10% FBS). VC = Vehicle control (50% Saline). Data shown as mean and standard deviation, n=5 for each group. **A:** VC treatment and 50% ARDS BAL treatment have no significant effect on AM viability (repeated measures ANOVA p=0.57). **B:** VC treatment and 50% ARDS BAL treatment have no significant effect on AM apoptosis (repeated measures ANOVA p=0.15).

**
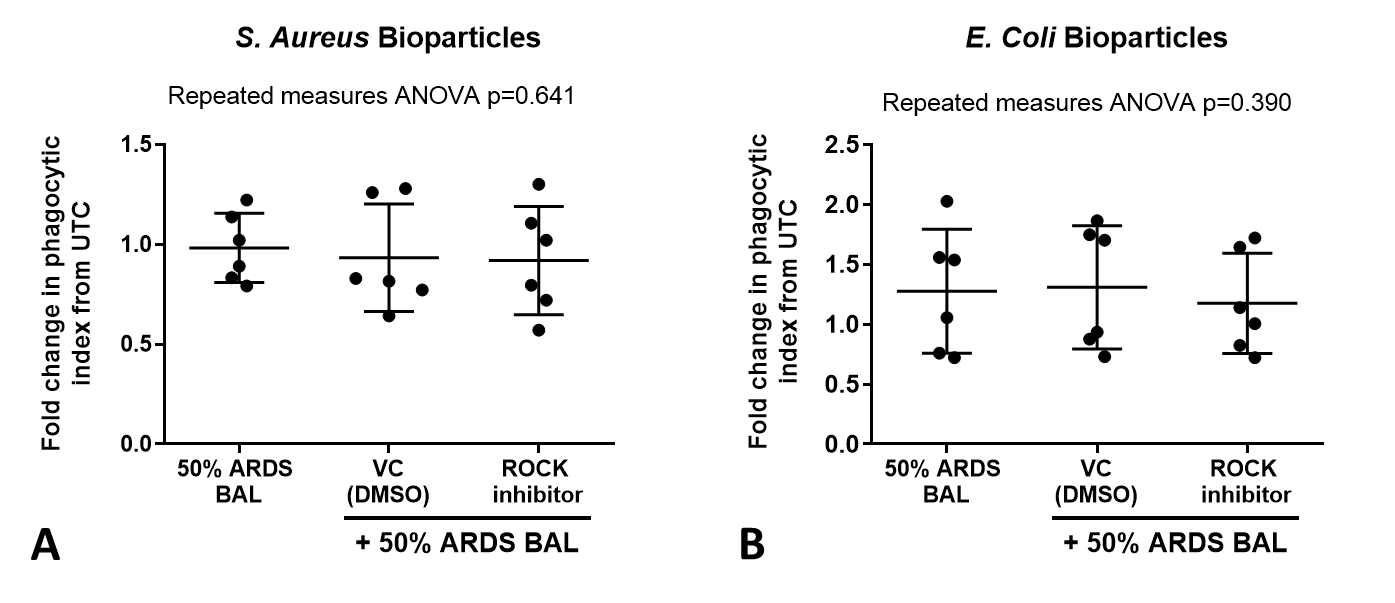
**

**Supplemental Figure 5: Effect of ARDS BAL and ROCK-inhibitor treatment on alveolar macrophage phagocytosis**

UTC = Untreated control. VC = Vehicle Control for ROCK-inhibitor (Dimethyl Sulfoxide [DMSO] at 1:50,000 dilution). ROCK-inhibitor = 200nM Y-27632 dihydrochloride; Rho-associated protein kinase inhibitor. 50% Saline acted as vehicle control for 50% ARDS BAL treatment. Data shown as mean and standard deviation, corrected to fold change in phagocytic index from UTC. Statistical analysis by repeated measures ANOVA. A: Addition of VC or ROCK-inhibitor to 50% ARDS BAL mixture had no significant effect on AM phagocytosis of *S. Aureus* pHrodo® bioparticles (repeated measures ANOVA p=0.641). B: Addition of VC or ROCK-inhibitor to 50% ARDS BAL mixture had no significant effect on AM phagocytosis of *E. coli* pHrodo® bioparticles (repeated measures ANOVA p=0.390).


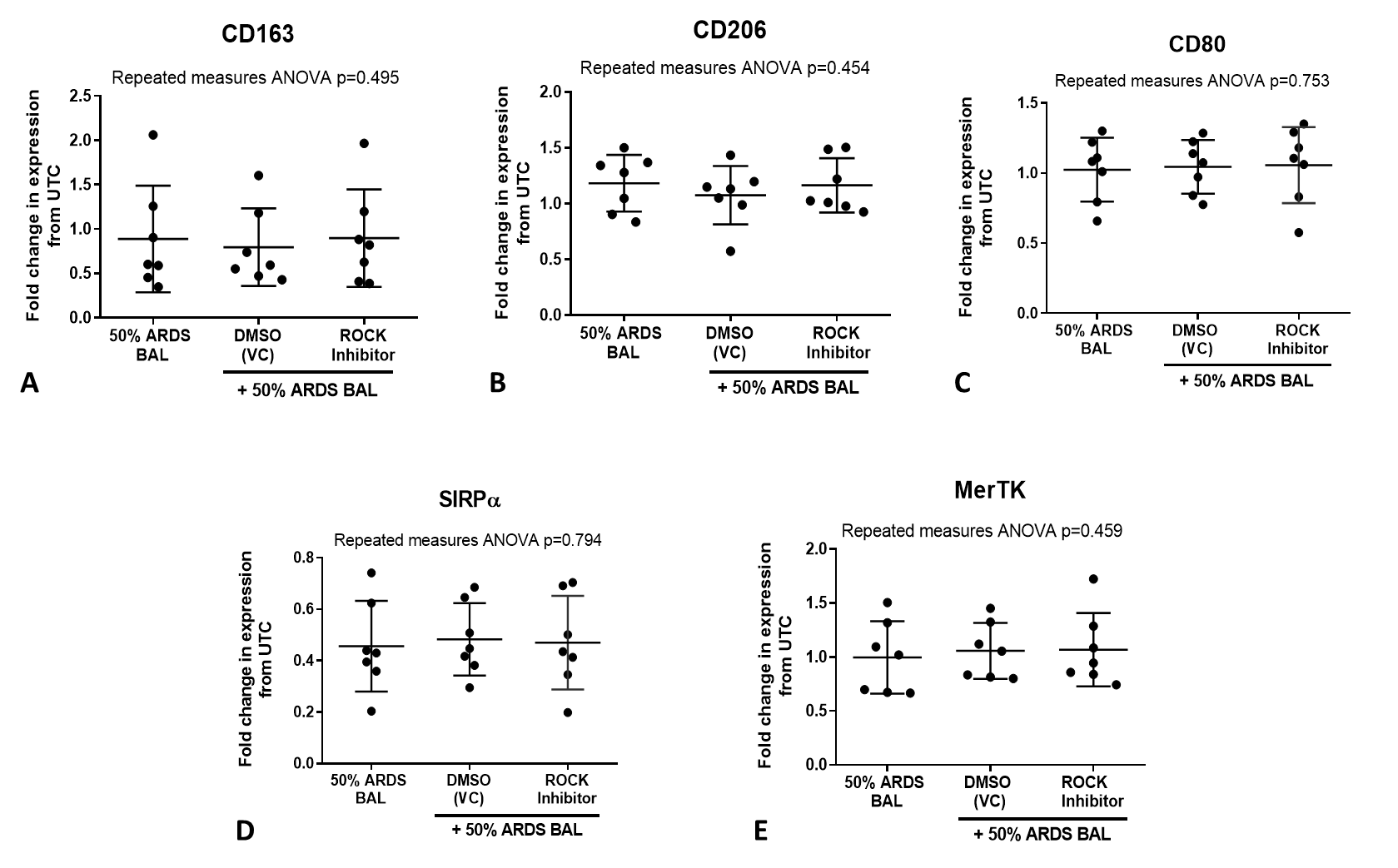


**Supplemental Figure 6: Effect of ROCK-inhibitor on alveolar macrophage surface receptor expression**

VC = Vehicle Control (Dimethyl Sulfoxide [DMSO] at 1:50,000 dilution). ROCK-inhibitor = 200nM Y-27632 dihydrochloride; Rho-associated protein kinase inhibitor. MerTK = Mer receptor tyrosine kinase. SIRPα = Signal regulatory protein alpha. Statistical analysis by paired t-test, n=7 for all groups. **A-E:** Addition of ROCK-inhibitor to 50% ARDS BAL treatment had no significant effect on AM surface expression of CD163, CD206, CD80, SIRPα and MerTK, compared to treatment with VC + ARDS BAL (p>0.05 for all) or ARDS BAL alone (repeated measures ANOVA p>0.45 for all).

| **Anti-Human Antibody** | **Clone** | **Fluorophore** | **Manufacturer** | **Isotype Control** | **Concentration** |
| --- | --- | --- | --- | --- | --- |
| CD206 | 19.2 | APC | BD Biosciences | Mouse IgG1κ | 1 : 50 |
| CD80 | L307.4 | PE | BD Biosciences | Mouse IgG1κ | 1 : 50 |
| CD163 | GHI/61 | FITC | BD Biosciences | Mouse IgG1κ | 1 : 50 |
| MerTK | 125518 | APC | R&D Systems | Mouse IgG2B | 1 : 50 |
| SIRPα (CD163) | 15-414 | FITC | ThermoFisher | Mouse IgG1κ | 1 : 50 |

**Supplemental Table 1: Flow cytometry antibody staining panels to assess alveolar macrophage surface marker expression**

FITC: Fluorescein isothiocyanate. APC: Allophycocyanin. PE: Phycoerythrin. Mer: Mer Receptor Tyrosine Kinase. SIRPα: Signal-Regulatory Protein Alpha. All Isotype control antibodies were purchased from ThermoFisher, except for Mouse IgG1aκ, which was from BD Biosciences.
